# Glycogen Content Regulates Peroxisome Proliferator Activated Receptor-∂ (PPAR-∂) Activity in Rat Skeletal Muscle

**DOI:** 10.1371/journal.pone.0077200

**Published:** 2013-10-17

**Authors:** Andrew Philp, Matthew G. MacKenzie, Micah Y. Belew, Mhairi C. Towler, Alan Corstorphine, Angela Papalamprou, D. Grahame Hardie, Keith Baar

**Affiliations:** 1 School of Sport, Exercise and Rehabilitation Sciences, University of Birmingham, Birmingham, United Kingdom; 2 Neurobiology, Physiology and Behavior, University of California Davis, Davis, California, United States of America; 3 Division of Cell Signalling & Immunology, University of Dundee, Dundee, United Kingdom; 4 Jacqui Wood Cancer Centre, University of Dundee, Dundee, United Kingdom; 5 Vivomotion, Greenhouse+, Dundee, United Kingdom; Virginia Tech, United States of America

## Abstract

Performing exercise in a glycogen depleted state increases skeletal muscle lipid utilization and the transcription of genes regulating mitochondrial β-oxidation. Potential candidates for glycogen-mediated metabolic adaptation are the peroxisome proliferator activated receptor (PPAR) coactivator-1α (PGC-1α) and the transcription factor/nuclear receptor PPAR-∂. It was therefore the aim of the present study to examine whether acute exercise with or without glycogen manipulation affects PGC-1α and PPAR-∂ function in rodent skeletal muscle. Twenty female Wistar rats were randomly assigned to 5 experimental groups (n = 4): control [CON]; normal glycogen control [NG-C]; normal glycogen exercise [NG-E]; low glycogen control [LG-C]; and low glycogen exercise [LG-E]). Gastrocnemius (GTN) muscles were collected immediately following exercise and analyzed for glycogen content, PPAR-∂ activity via chromatin immunoprecipitation (ChIP) assays, AMPK α1/α2 kinase activity, and the localization of AMPK and PGC-1α. Exercise reduced muscle glycogen by 47 and 75% relative to CON in the NG-E and LG-E groups, respectively. Exercise that started with low glycogen (LG-E) finished with higher AMPK-α2 activity (147%, p<0.05), nuclear AMPK-α2 and PGC-1α, but no difference in AMPK-α1 activity compared to CON. In addition, PPAR-∂ binding to the CPT1 promoter was significantly increased only in the LG-E group. Finally, cell reporter studies in contracting C2C12 myotubes indicated that PPAR-∂ activity following contraction is sensitive to glucose availability, providing mechanistic insight into the association between PPAR-∂ and glycogen content/substrate availability. The present study is the first to examine PPAR-∂ activity in skeletal muscle in response to an acute bout of endurance exercise. Our data would suggest that a factor associated with muscle contraction and/or glycogen depletion activates PPAR-∂ and initiates AMPK translocation in skeletal muscle in response to exercise.

## Introduction

Skeletal muscle glycogen content determines an individual's capacity to sustain long duration exercise [1]. In the absence of glycogen, muscle increases its metabolic reliance on free fatty acid (FFA) oxidation [2]. This metabolic flexibility indicates that an inverse relationship exists between glycogen content and lipid utilization in skeletal muscle, which might contribute to the increased capacity for fat oxidation in skeletal muscle following endurance training [3].

The glycogen content of skeletal muscle has also been shown to affect the post exercise transcriptional response [4]. Notably, reducing glycogen content prior to exercise increases the activation of key genes involved in mitochondrial biogenesis and lipid oxidation, suggesting that glycogen content regulates the magnitude of skeletal muscle adaptation to endurance exercise [4], [5], [6]. Accordingly, Hansen *et al*., [7] hypothesised that training with depleted glycogen would result in greater muscle adaptation than the equivalent training with normal or high glycogen levels. Over a 10-week period, human volunteers performed single leg kicking exercise every day (normal glycogen) or twice every other day (low glycogen), with the second session performed with low muscle glycogen. Following the training period there was a greater increase in time to exhaustion in the low glycogen group and increased activity of the β-oxidation and TCA enzymes (3-hydroxyacyl-CoA dehydrogenase (HAD) and citrate synthase (CS)) [7].

Following a similar training design, we [8] and others [9] have demonstrated that training in a low glycogen state also leads to increased FFA oxidation in trained cyclists and increases the mobilization and utilization of intra-muscular triglycerides (IMTG) [8]. Given this observation, it has been hypothesized that alterations in FFA availability, potentially as a consequence of IMTG breakdown, might function as a signal to initiate molecular adaptation [3]. Two potential targets for circulating FFA are the peroxisome proliferator activated receptor family of nuclear receptors (PPAR) and the transcriptional coactivator PGC-1α (peroxisome proliferator activated receptor-γ coactivtor-1α) [10]. Two PPAR isoforms, PPAR-α and PPAR-∂, are expressed in skeletal muscle and have been shown to control the expression of a variety of genes involved in FFA utilization and oxidation [11]. Mice with transgenic expression of an activated form of PPAR-∂ display increased expression of genes involved in lipid metabolism and present resistance to high-fat induced obesity [12] suggesting that PPAR-∂ might be the molecular target activated by low glycogen training.

PGC-1α is a transcriptional co-activator known to target transcription factors regulating mitochondrial metabolism, fatty acid oxidation enzymes, and angiogenesis in skeletal muscle [13]. PGC-1α function is highly sensitive to exercise, and has been suggested to co-ordinate the mitochondrial post-exercise transcriptional response [13], although this process can occur independently of PGC-1α in both rodent [14] and human [15], [16] exercise models. Consistent with this concept, PGC-1α muscle-specific transgenic mice display a muscle energetic profile similar to highly trained athletes [17] and an increased expression of genes known to be involved in lipid utilization and oxidation [17].

It was therefore the aim of the present study to examine whether (1) acute exercise alters PGC-1α/PPAR-∂ activity or localization in skeletal muscle, and (2) whether manipulating skeletal muscle glycogen content alters the exercise-induced PGC-1α/PPAR-∂ response.

## Materials and Methods

### Ethical approval

All procedures were approved by the University of Dundee research ethics committee and performed under UK Home Office project licence number 60/3441.

### Materials

All reagents were from Sigma Aldrich (UK) unless otherwise stated. Total AMPK α1/α2 antibodies were described previously [18]. Antibodies against PGC-1α were obtained from Millipore (AB3242), Histone H2B from AbCam (ab9408), LDH from Sigma (L7016). (9212) were from Cell Signaling (Cell Signaling, Danvers, MA).

### Exercise protocol

**Figure 1:**
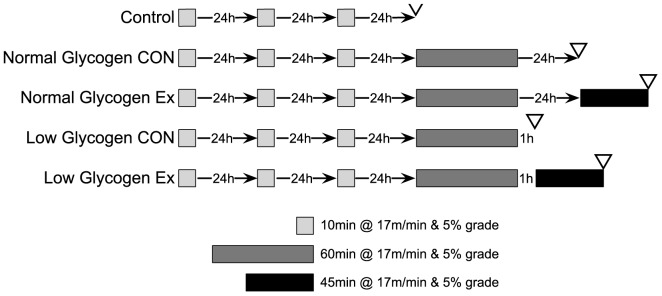
Schematic of the experimental design. Skeletal muscle glycogen content was manipulated using pre-exercise and varying periods of recovery, prior to a main 45 min exercise trial. Five groups performed 3-days acclimatization to treadmill running. Following which, the CON group was sacrificed 24 h after the final acclimatization trial, the normal glycogen control (NG-C) ran for 1 hour and then rested 23 h before sacrifice, the normal glycogen exercise (NG-E) performed a subsequent 45 min run 23 h after the initial 1 hour run, the low glycogen control (LG-C) was collected 1 h after the initial 1 hour run, and the low glycogen exercise group (LG-E) was collected immediately following a 45 min run commenced 1 hour after the initial 1 hour trial. All experiments took place in the morning to avoid significant alterations in diet and diurnal variation in metabolic responses.

Twenty, female Wistar rats ∼200 g (Charles River Laboratories, Tranent, UK) were used for the study. Running experiments took place on a 3-lane motorized treadmill (Eco 3/6 Treadmill; Columbus Instruments, Ohio, USA) with the gradient set at 5%. The exercise protocol is detailed in Figure 1. Briefly, rats (n = 4) were separated into five groups consisting of a no exercise control (CON), normal glycogen control (NG-C), normal glycogen exercise (NG-EX), low glycogen control (LG-C) and low glycogen exercise (LG-EX). Each rat received three separate habituation runs (Day 1, 2 and 3) at 17 m.min^-1^ lasting 10 mins prior to commencing the main experimental trial. The main experimental trial consisted of a 60 min exercise bout at 17 m.min^-1^ followed by a second 40 min exercise bout 23 h later (NG-E) or 1 h later (LG-E). The NG-C and LG-C controls were collected following the 23 h or 1 hr recovery period, before the start of the second exercise bout to allow for assessment of pre-exercise glycogen levels in the NG-E and LG-E groups. Immediately upon completion of exercise, rats were terminated via cervical dislocation following concussion, the gastrocnemius muscles were dissected and snap frozen in liquid nitrogen.

### Tissue collection and homogenization

Tissue was powdered under liquid nitrogen using a mortar and pestle. 50 mg of gastrocnemius muscle was homogenized in 10-fold mass excess of ice cold sucrose lysis buffer (50 mM Tris pH 7.5, 250 mM Sucrose, 1 mM EDTA, 1 mM EGTA, 1% Triton X 100, 50 mM NaF, 1 mM NaVO_4_ Na_2_(PO_4_)_2_ and 0.1% DTT) using a hand held homogenizer (Pro 200, PRO scientific INC, USA). The homogenate was briefly vortexed and centrifuged at 4°C/8000xg for 10 mins to remove insoluble material. Protein concentrations were determined using the DC protein assay (Bio-Rad, Hercules CA).

### Glycogen measurements

Powdered muscle (30 mg) was hydrolyzed in 250 µl of 2 N HCl by heating at 95°C for 3 h. The solution was neutralized with 250 µl 2 N NaOH, and the resulting free glycosyl units were assayed spectrophotometrically using a hexokinase-dependent assay kit from Amresco against glucose standards of known concentrations [19].

### Western blot procedures

Equal aliquots of protein were boiled for 5 minutes in 1× Laemmli sample buffer and separated on 7.5 or 10% gels by SDS-polyacrylamide gel electrophoresis (PAGE). Following electrophoresis, proteins were transferred for 1 hour at 100 V to a Protran nitrocellulose membrane (Whatman, Dassel, Germany), blocked for 1 hour in 3% milk in TBST (Tris-buffered saline +0.1% Tween), and then incubated over night at 4°C with appropriate primary antibody in TBST at 1∶1000. The following day, membranes were washed 3× in TBST before incubation for 1 hour at room temperature with peroxidase-conjugated secondary antibodies in TBST at 1∶10,000 (Perbio Science, Cramlington, UK). Antibody binding was detected using an enhanced chemiluminescence HRP substrate detection kit (Millipore, Watford, UK). Imaging and band quantification were carried out using a ChemiGenius Bioimaging Gel Doc System (Syngene, Cambridge, UK).

### AMPK activity assay

Approximately 50 mg of GTN muscle was suspended in 250* µ*l homogenization buffer (50 mm Tris/HCl, 0.25 m mannitol, 50 mm NaF, 5 mm sodium pyrophosphate, 150 mm NaCl, 5* µ*gml−1 soybean trypsin inhibitor, 1 mm DTT, 0.1 mm PMSF, 1% (v/v) Triton X-100) and homogenized using a hand-held polytron. The samples were left to rotate at 4°C for 30 mins and then cell debris was pelleted by centrifugation at 17,600 *g* for 5 min. The supernatants were removed and protein concentration determined. Sheep anti-AMPK α1 or α2 antibodies (5* µ*g) were used to immunoprecipitate AMPK from 200* µ*g of muscle lysate for 2 h at 4°C. The immunoprecipitates were washed in homogenization buffer and then resuspended in assay buffer (50 mm Hepes, 1 mm DTT, 0.02% (v/v) Brij-35). AMP, [32−*γ* P]-ATP (200 cpm pmol−1) and the peptide substrate (AMARAASAAALARRR) were added to the immunoprecipitate at a final concentration of 200 µm. The assays were carried out for 15 min at 30°C and terminated by applying 30* µ*l of the reaction mixture to P81 papers (Whatman; Maidstone, UK). ATP incorporation into the peptide substrate was inhibited by placing the filter paper in 1% orthophosphoric acid. Phosphate incorporation into the peptide was measured as previously described [20].

### Nuclear protein isolation

Nuclear and cytosolic fractions were isolated as previously described [21]. Briefly, 50 mg GTN muscle was homogenized using a commercially available kit (78835: NE-PER; Thermo Scientific) according to the manufacturers instructions, with the addition of the complete protease inhibitor mixture (11697498001; Roche Applied Science). Integrity of nuclear fractions was confirmed by immunoblotting for the cytosolic enzyme, lactate dehydrogenase (LDH, L7016; Sigma) and the nuclear protein, histone H2B (ab9408; AbCam).

### Chromatin Immunoprecipitation (ChIP) assay

Chromatin immunoprecipitation experiments were performed using a commercially available kit according to the manufacturer's instructions (Millipore, CA, USA, Cat: 17–295). Briefly, 200 µg of powdered GTN muscle was rotated end-over-end for 10 mins in 10 ml ice-cold 1% high-grade formaldehyde/PBS. Muscle samples were homogenized in 250 µL SDS lysis buffer using a hand-held homogenizer (Pro 200, PRO scientific INC, USA). DNA was sheared using a hand-held sonicator on ice and the resulting DNA/protein complexes immunoprecipitated overnight using a mouse monocolonal PPAR-∂ antibody (R&D Systems INC, Cat: PP-K9436-10) or a goat anti-mouse IgG as control (Thermo Scientific, Cat: 31430). The following day, samples were reversed crosslinked and DNA extracted. To determine PPAR-∂ binding to the CPT1 promoter, primers were designed to flank a 285 bp region of the CPT1 promoter. The primers used were (FWD 5′-CTGGAGAGGAATGGGACAAC-3′) and (REV 5′-ATTGGGGTGGAGAAAACAGA-3′) with the RT-qPCR cycle consisting of an initial 95°C for 5 min, followed by 50 cycles of 95°C for 15 s, 56°C for 15 s and 72°C for 30 s.

### Expression plasmids and reporter systems

The 4xACO plasmid was previously described by He and colleagues [22] and was purchased from Addgene (16533). Briefly this plasmid contains 4 copies of the prototypic PPAR-responsive element (PRE) taken from the acyl-CoA oxidase (ACO) gene promoter, which contains two copies of the core binding sequence AGGTCA separated by one base pair. The pCMX-PPARβ/∂ expression vector was generously provided by Professor Dan Kelly, Sandford-Burnham Medical Research Institute, Lake Nona, USA and has been described in full [23]. The Renilla luciferase expression vector (RLuc) was purchased from Promega (Madison, WI).

### C2C12 transient cell transfections

C2C12 myoblasts were obtained from the ATCC. Transient transfection of PPAR-∂, ACO, and RLuc were performed on sub-confluent C2C12 myoblasts using lipofectamine 2000 reagent (Invitrogen, Carlsbad, CA) as previously described [24]. 1×10^5^ cells were seeded on 6-well plates. The next day, myoblasts were transfected with 0.25 µg PPARδ, 2.00 µg ACO, and 0.50 µg RLuc, complexed with lipofectamine in 500 µl OptiMEM transfection media (Invitrogen, Carlsbad, CA) and added to each well. The next day, cells were washed twice with PBS and placed in differentiation media (High glucose DMEM, 2% HS, 1% Pen/strep).

### Cell glycogen depletion and contraction

Following transfection, cells were maintained in growth media for 24 h before shifting to differentiation media (DM; High glucose DMEM, 2% HS, 1% Pen/strep). After four days, fully differentiated myotubes were washed once with DPBS and transferred to glucose free DMEM media (LG) or maintained in high glucose DM. 24 hrs later, media was replenished for 3 or 6 hours and the cells collected, or the cells were stimulated for 3 h (10 Hz, 0.4 s contraction, 3.6 s delay) using a pulse width of 0.3 ms and pulse amplitude of 40 V as previously described [24]. Following treatment, the media was aspirated; the cells were rinsed in PBS, and collected in 500 µl of passive lysis buffer (PLB; Madison, WI) while rocking for 15 min. The lysates were centrifuged at 4°C for 3 minutes at 14,000 g to remove debris. The supernatant (20 µl) was transferred to a 96-well plate and promoter activity was assayed using the Dual Luciferase Reporter 1000 Assay System (Promega, E1980). PPAR-∂ activity was determined by normalizing firefly luciferase activity (ACO-luciferase) to renilla luciferase (SV40-renilla). Absolute renilla was used to determine cell viability and transfection efficiency and was not different between treatment groups.

### Statistical analysis

One-way analysis of variance ANOVA (BrightStat.com) and Tukey honestly significant difference posthoc test were used to determine differences in glycogen content, PPAR-∂ activity, AMPKα1/α2 activity, protein localization and phosphorylation between groups. Values are displayed as mean ± SEM, with statistical significance set at 0.05.

## Results

### Glycogen content

**Figure 2:**
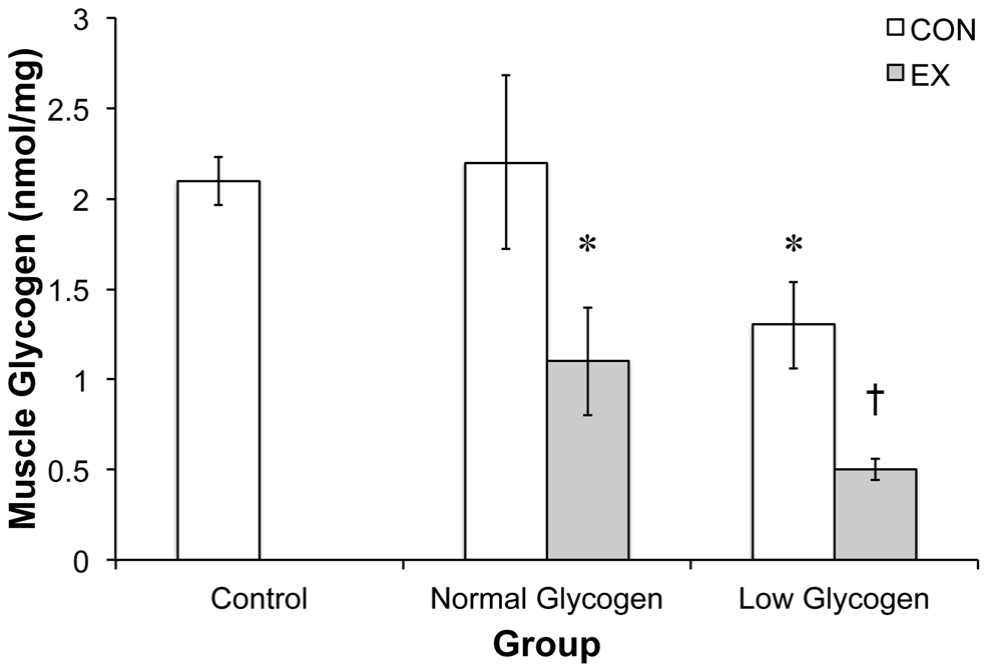
Glycogen levels in the 5 experimental groups. Glycogen was determined after hydrolysis of 30* indicates significantly different than CON and † indicates significantly different than NG-E and LG-C (p<0.05; n = 4).

The exercise protocol successfully reduced GTN glycogen content (Figure 2). As expected, the 23 h recovery period restored glycogen in the NG-C group without super-compensation, resulting in the NG groups conducting the second exercise bout with glycogen concentration similar to the no exercise control (CON) group (2.1±0.1 to 2.2±0.4 nmol.mg.wet wt). Following the 40 min exercise bout, we observed a 48% reduction in the NG-E group compared to NG-C (2.2±0.4 to 1.1±0.2 nmol.mg.wet wt). In contrast, the LG-C group had 40% less glycogen content than the CON group (2.1±0.1 to 1.3±0.3 nmol.mg.wet wt). At the end of the second exercise bout, the LG-E group had a 75% reduction in total glycogen compared to the CON group (1.3±0.3 to 0.5±0.06 nmol.mg.wet wt).

### AMPK α1 activity

**Figure 3:**
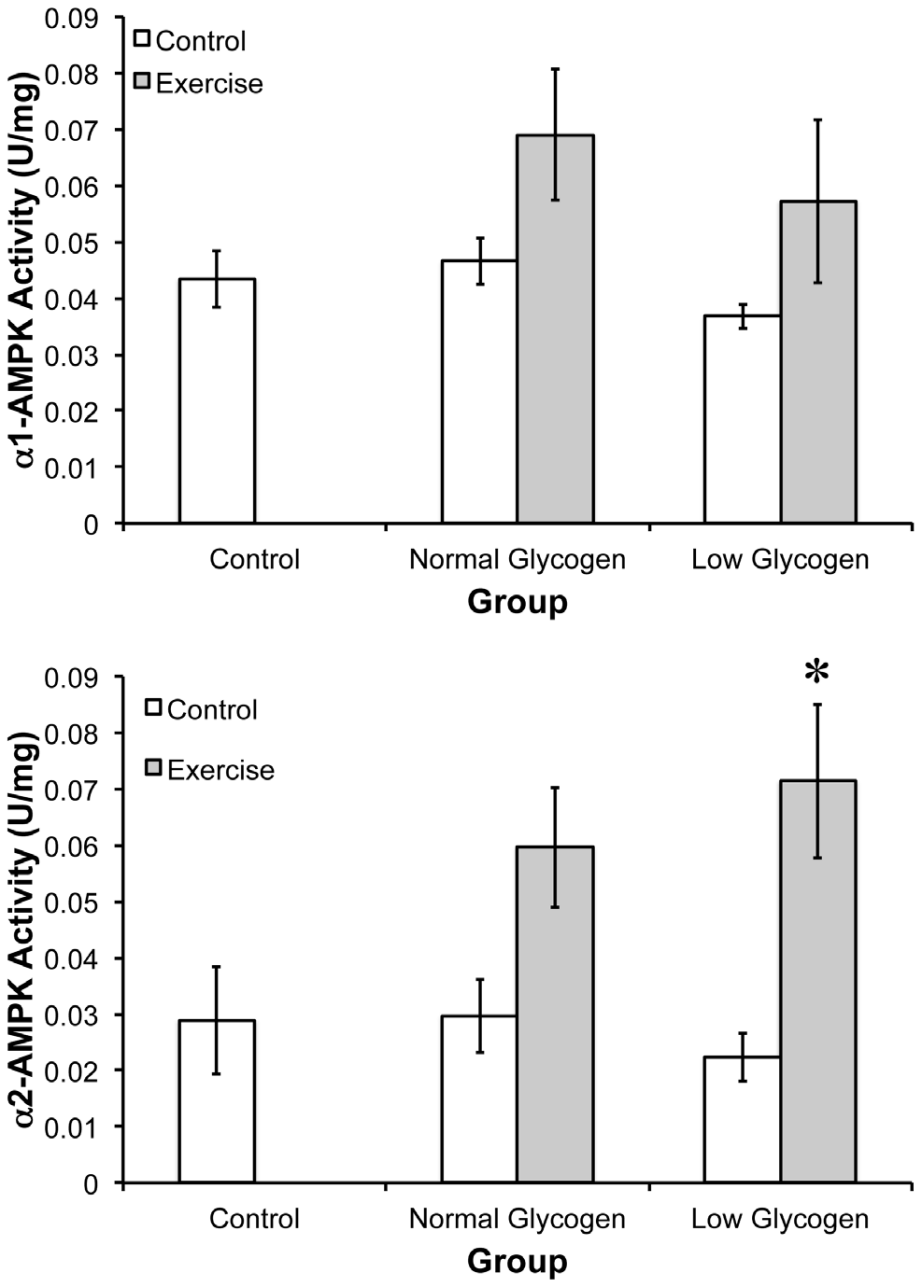
AMPK activity following exercise in a normal or low glycogen environment. The activity of (A) α1-AMPK and (B) α2-AMPK was determined in each of the 5 groups by IP kinase activity assay. * indicates significantly different than control (p<0.05; n = 4).

AMPK α1 activity was not different between the CON, NG-C (0.04±0.002 to 0.4±0.001 Units mg/AMPK), or LG-C groups (CON 0.04±0.002 to LG-C 0.3±0.001 Units mg/AMPK; Figure 3A). AMPK α1 activity tended to increase following exercise in the NG-E group (CON 0.04±0.002 to NG-E 0.07±0.005 Units mg/AMPK) and the LG-E group (CON 0.04±0.002 to LG-E 0.05±0.006 Units mg/AMPK), but neither reached statistical significance.

### AMPK α2 activity

AMPK α2 activity was not different between the CON, NG-C (0.03±0.004 to 0.03±0.003 Units mg/AMPK), or LG-C groups (CON 0.03±0.004 to LG-C 0.02±0.001 Units mg/AMPK; Figure 3B). AMPK α2 activity tended to increase (106%; p = 0.127) following exercise in the NG-E group (CON 0.03±0.002 to NG-E 0.06±0.004 Units mg/AMPK). Given the suppression in AMPK α2 activity in the LG-C group, the change in AMPK α2 activity following the low glycogen exercise bout was 219% (LG-C 0.02±0.001 to LG-C 0.07±0.005 Units mg/AMPK).

### PGC-1α, AMPKα1 and AMPKα2 nuclear abundance

**Figure 4:**
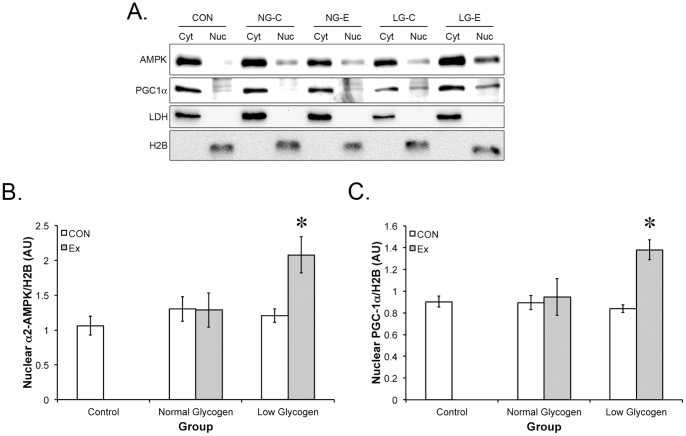
Signaling in response to exercise in a normal or low glycogen environment. The localization (A) of α2-AMPK and PGC-1α was determined following exercise in a normal or glycogen depleted state. Nuclear abundance of (B) α2-AMPK and (C) PGC-1α was determined relative to histone H2B. * indicates significantly different than control (p<0.05; n = 4).

PGC-1α nuclear abundance increased in the LG-E group by 52% but was unchanged in any other group (Figure 4). In a similar manner, AMPKα2 nuclear abundance increased in the LG-E group by 95%, with no change in AMPKα2 nuclear abundance was observed in any other group. In contrast to AMPKα2, AMPKα1 nuclear translocation was not observed with exercise.

### Skeletal muscle PPAR-∂ activity

**Figure 5:**
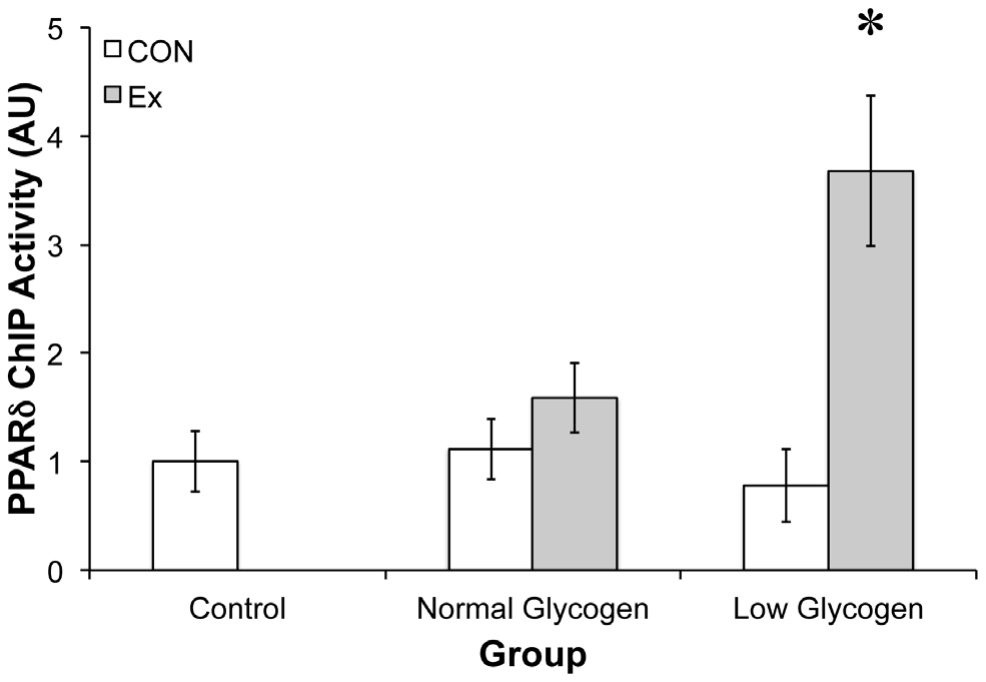
PPAR-∂ activity in response to exercise in a normal or low glycogen environment. Post-exercise skeletal muscle activity of PPAR-∂ was determined by chromatin immunoprecipitation (ChIP) assay followed by qRT-PCR using primers spanning the PPAR-∂ binding region in the CPT-1 promoter. * indicates significantly different than control (p<0.05; n = 4).

PPAR-∂ activity, as assessed by PPAR-∂ binding to the CPT1 promoter (Figure 5), was observed to increase ∼400% in the LG-E group immediately after exercise, with no changes observed in any of the other groups.

### Cell PPAR-∂ activity

**Figure 6:**
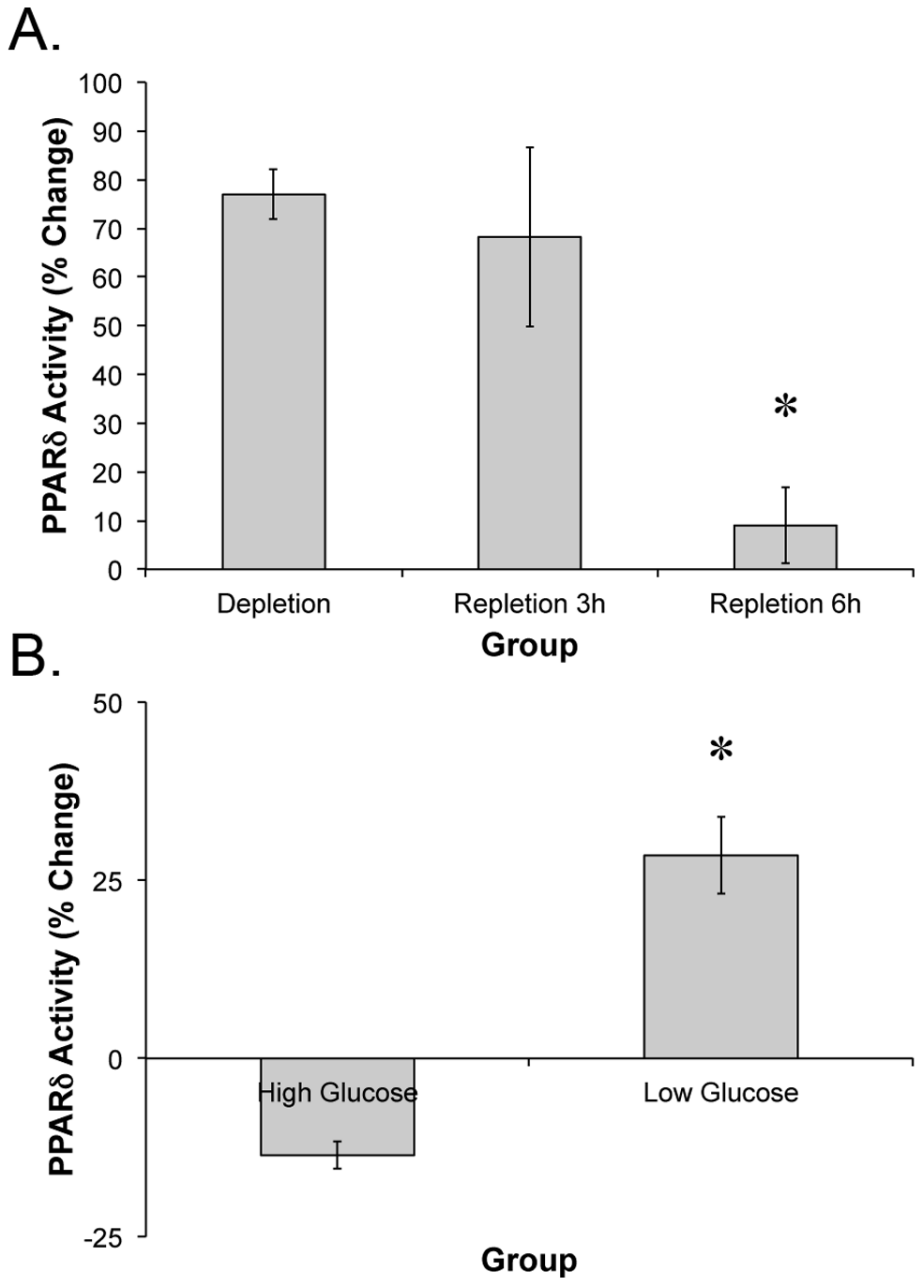
PPAR-∂ activity in response to decreased glucose availability and electrical stimulation. The *in vitro* activity of PPAR-∂ was determined by measuring 4xACO-luciferase expression following (A) glycogen depletion and repletion, or (B) electrical stimulation in a glucose depleted or glucose rich environment. * indicates significantly different than control (p<0.05; n = 6 three independent replicates).

In cell culture experiments, glycogen depletion increased PPAR-∂ activity 76%, suggesting a direct effect of glycogen content on PPAR-∂ activity (Figure 6). In agreement with this hypothesis, glucose refeeding was characterized by a gradual suppression of PPAR-∂ activity, returning to basal activity levels after 6 hs. When contraction was initiated in a low glucose state, PPAR-∂ activity increased 28%. In contrast, performing contraction in a normal glucose media resulted in a 13% suppression of PPAR-∂ activity compared to basal control conditions.

## Discussion

In the present study, we assessed the effect of muscle glycogen, on post exercise signaling mechanisms that may regulate lipid utilization in skeletal muscle. Importantly, to achieve this, we manipulated glycogen levels via prior exercise, rather than nutritional intervention. This is an important point, as this approach allowed us to investigate the affects of glycogen depletion and subsequent alteration in endogenous substrate availability, rather than artificially manipulating substrate content, which may have directly altered cellular signaling events. The validity of this approach is seen in the fact that the LG-E group began exercise with 52% of the glycogen of the CON group and by the end of exercise displayed a 75% decrease in glycogen content. The physiological reduction in muscle glycogen content that we report here is similar to that reported in *ex vivo* models [25] and following exercise *in vivo* [26]. With regard to the exercise protocol, it should also be noted that we aimed to compare depleted vs replenished glycogen content, rather than high vs low, as glycogen super-compensation has previously been shown to alter substrate metabolism and intracellular signaling during and following acute exercise [26].

The principal finding of the present study was the observation that PPAR-∂ transcriptional activity is sensitive to the combined effect of skeletal muscle contraction and glycogen depletion. We observed an increase in PPAR-∂ binding to the CPT-1 promoter when running exercise was conducted in a glycogen-depleted state (LG-E). Further, to support our skeletal muscle observations, we present cell-based evidence that PPAR-∂ activity is directly sensitive to glucose availability and glycogen content, and restoring glucose availability suppresses PPAR-∂. Collectively, this data has important implications for the adaptive response of skeletal muscle to endurance-type exercise.

Unlike other, more characterized PPAR family members (PPAR-γ and PPAR-α), there is limited information regarding the role of PPAR-∂ in skeletal muscle metabolic regulation. PPAR-∂ is the most abundant PPAR in skeletal muscle and has been shown to be enriched in oxidative type I fibers [27]. Given the association of PPAR-∂ expression with skeletal muscle oxidative capacity, it is not surprising that both acute [28], [29] and chronic [30], [31] exercise increases PPAR-∂ mRNA in both rodent and human skeletal muscle. These observations have therefore led to the suggestion that PPAR-∂ activity is under the control of an exercise-derived factor [3], [11], however the precise mechanism for PPAR-∂ induction during muscle contraction remains unknown.

One possible mechanism by which exercise may trigger PPAR-∂ activation is via the liberation of FFAs, which are PPAR ligands, from adipose and intramuscular stores. Indeed, Fyffe and colleagues recently demonstrated that long-chain FFA [C16:0, C16:1, C18:0 and C18:1] were able to occupy a recombinant PPAR-∂ protein ligand binding domain in a ratio of 3∶2∶1∶4, with ligand binding thought to stabilize the protein in an active state [32]. Given that endogenous FFA of similar chain length (palmitate [C16]; palmitoleate [C16:1]; and oleate [C18:1]) have been shown to have diverse physiological functions in skeletal muscle [5], determining whether such FFA, or species derived from these FFA are the endogenous PPAR-∂ ligand is a key future question to be addressed.

One interesting caveat with regard to FFA as an *in vivo* ligand for PPAR-∂ during exercise is the recent study by Watt and colleagues, which demonstrated that treatment with the anti-lipolytic drug nicotinic acid (causing a reduction in FFA availability) actually increased PPAR-∂ mRNA expression following acute exercise [29]. Further, treatment of C2C12 myotubes with long-chain FFA failed to increase PPAR-∂ expression [33]. Thus, it seems that we are still some way from understanding the endogenous ligand for PPAR-∂ in skeletal muscle. Given the disparity between the activity of PPAR-∂ target genes and PPAR-∂ gene expression, it may also be that the activity of PPAR-∂ (i.e. cellular location and binding of PPAR-∂ to target gene promoters), rather than increased protein or mRNA abundance is the important physiological event regarding PPAR-∂ activity. To support this hypothesis, the data herein provides the first evidence that contraction in a glycogen depleted state increases PPAR-∂ activity in skeletal muscle.

Given our observation that glycogen depletion was not sufficient to increase PPAR-∂ activity *in vivo*, it would appear that a contraction-derived signal is required to activate PPAR-∂. Little is known regarding modes of post-translational regulation of PPAR-∂ activity. However, phosphorylation has been reported as a mode of regulation for both PPAR-γ and PPAR-α [34]. Exercise in a glycogen-depleted state is known to result in increased activation of a host of cell energy sensing kinases [3], all implicated in mitochondrial adaptation to exercise. Consistent with previous research, we observed increased AMPK α2 activity and nuclear abundance following exercise in the LG-E group. These findings are in agreement with data in both rodents [25] and humans [26], [35], suggesting that contraction mediated activation and relocalization of AMPK is sensitive to skeletal muscle glycogen content. Overlap in function between AMPK and PPAR-∂ has been reported by Kramer and colleagues who demonstrated that the PPAR-∂ agonist GW501516 (GW) increases the activity of both AMPK and PPAR-∂ in human primary myotubes [36]. In contrast, *in vivo* GW does not activate AMPK and only marginally increases target gene expression [37]. When GW treatment is combined with the AMPK activator AICAR, target gene expression increases 2-3-fold more than with GW alone [37]. This notion is also supported by the recent work by Gan and coworkers who demonstrated that PPAR-∂, but not PPAR-α, interacted with AMPK to synergistically increase lactate dehydrogenase (LDH) gene transcription [38]. Collectively this data suggests that AMPK and PPAR-∂ may function together to increase the expression of enzymes important in fatty acid oxidation. Further, our data suggest that low glycogen drives AMPK into the nucleus possibly as a result of less direct binding of AMPK to glycogen. However, the exact mechanism and the regulatory steps involved remain to be determined.

To confirm the effects of glycogen depletion on PPAR-∂ activity, we used an *in vitro* system to determine the effects of removing glucose from the medium followed by glucose refeeding or electrical stimulation (contraction). In accordance with our *in vivo* finding, PPAR-∂ was activated by removal of glucose and inactivated 6 hours after glucose has been added back to the medium. Extending these findings, we show that contraction further increases PPAR-∂ activity in a low glucose environment, whereas in a high glucose environment contraction decreased PPAR-∂ activity. This finding is strikingly similar to β-HAD levels in trained humans following 3 weeks of training in either a high or low glycogen state [8], suggesting that PPAR-∂ activity may underlie some of the beneficial effects of low glycogen exercise on fatty acid metabolism.

Another novel finding of the present study was the observation that PGC-1α nuclear translocation only occurred in the LG-E group. Terada and Tabata were the first to observe an increase in skeletal muscle PGC-1α nuclear abundance following exercise [39]. Complimentary studies from the same group also showed a clear association between exercise duration/intensity, the magnitude of glycogen depletion, and the amount of PGC-1α nuclear induction in exercised rat skeletal muscle [40]. Subsequent research has shown that the nuclear import of PGC-1α appears to be an initiator of the mitochondrial biogenetic program [41], and that this process also occurs in humans following acute endurance [42] and high intensity [43] exercise. However, even though an increase in skeletal muscle nuclear PGC-1α following exercise is generally accepted, little is known mechanistically about the regulation of this process. Future research should focus on identification of the factors involved in PGC-1α nuclear translocation following exercise.

The present study demonstrates the sensitivity of cellular signaling to glycogen content. However, important limitations in the study design should be acknowledged. We were careful to manipulate glycogen content via exercise, and not nutrition. In doing so, the LG-E group therefore had successive bouts of exercise compared to the N-GE group who had a significant rest period. To minimize the effect of the rest period, we added two additional control groups (NG-C and LG-C) so that we could understand cellular signaling prior to the second bout of exercise. Nevertheless, there remains the possibility that the successive bout of exercise in the LG-E group may have produced a greater exercise stimulus, which may have contributed to the exercise adaptation we observed. However, until a genetic or pharmacological method is developed to specifically manipulate glycogen content independent of diet or exercise, these study limitations will remain.

## Conclusion

In conclusion, we report for the first time that exercise and glycogen regulate the activity of PPAR-∂ in skeletal muscle. During exercise in a glycogen-depleted state we observed increased binding of PPAR-∂ to the CPT1 promoter, indicative of increased transcriptional activation of PPAR-∂. Further, we also report that PPAR-∂ activity *in vitro* is sensitive to glucose availability and glycogen content. In addition, we report that the nuclear translocation of AMPK and PGC-1α is sensitive to glycogen content. This observation has important implications for mitochondrial adaption to exercise and provides a potential mechanism for elevated FFA oxidation in glycogen depleted skeletal muscle. These observations could potentially underlie the improved performance seen in recreational [7], [8] and elite [44] athletes who incorporate low glycogen training into their preparation for long distance events. Further research should aim at determining the cellular cues by which glycogen depletion increases PGC-1α/AMPK/PPAR-∂ activity and the implications for this with regard to skeletal muscle adaptation following exercise.
